# Antioxidant Cardioprotection against Reperfusion Injury: Potential Therapeutic Roles of Resveratrol and Quercetin

**DOI:** 10.3390/molecules27082564

**Published:** 2022-04-15

**Authors:** Ramón Rodrigo, Catalina Retamal, Denisse Schupper, Diego Vergara-Hernández, Sarmistha Saha, Elisabetta Profumo, Brigitta Buttari, Luciano Saso

**Affiliations:** 1 Molecular and Clinical Pharmacology Program, Faculty of Medicine, Institute of Biomedical Sciences, University of Chile, Santiago P.O. Box 70111, Chile; catalinaretamal@ug.uchile.cl (C.R.); denisse.schupper@ug.uchile.cl (D.S.); diego.vergara.h@ug.uchile.cl (D.V.-H.); 2 Indian Institute of Technology Gandhinagar, Palaj, Gandhinagar 382055, Gujarat, India; sarmisthasaha.53@gmail.com; 3 Department of Cardiovascular, Endocrine-Metabolic Diseases, and Aging, Italian National Institute of Health, 00161 Rome, Italy; elisabetta.profumo@iss.it (E.P.); brigitta.buttari@iss.it (B.B.); 4 Department of Physiology and Pharmacology “Vittorio Erspamer”, Sapienza University of Rome, 00161 Rome, Italy; luciano.saso@uniroma1.it

**Keywords:** oxidative stress, reperfusion injury, antioxidants, resveratrol, quercetin, cardioprotection

## Abstract

Ischemia-reperfusion myocardial damage is a paradoxical tissue injury occurring during percutaneous coronary intervention (PCI) in acute myocardial infarction (AMI) patients. Although this damage could account for up to 50% of the final infarct size, there has been no available pharmacological treatment until now. Oxidative stress contributes to the underlying production mechanism, exerting the most marked injury during the early onset of reperfusion. So far, antioxidants have been shown to protect the AMI patients undergoing PCI to mitigate these detrimental effects; however, no clinical trials to date have shown any significant infarct size reduction. Therefore, it is worthwhile to consider multitarget antioxidant therapies targeting multifactorial AMI. Indeed, this clinical setting involves injurious effects derived from oxygen deprivation, intracellular pH changes and increased concentration of cytosolic Ca^2+^ and reactive oxygen species, among others. Thus, we will review a brief overview of the pathological cascades involved in ischemia-reperfusion injury and the potential therapeutic effects based on preclinical studies involving a combination of antioxidants, with particular reference to resveratrol and quercetin, which could contribute to cardioprotection against ischemia-reperfusion injury in myocardial tissue. We will also highlight the upcoming perspectives of these antioxidants for designing future studies.

## 1. Introduction

Cardiovascular disease is the most common cause of morbidity and mortality worldwide, with 17.9 million deaths every year, according to updated WHO data from the year 2017, which represents 31% of all registered deaths in the world [1]. Among cardiovascular diseases, stroke and acute myocardial infarct (AMI) are the main causes of death, the latter being responsible for around 9 million deaths each year [2]. AMI is produced by either partial or complete occlusion of the coronary arterial circulation. It is mainly caused by atheromatous plaques that are vulnerable to rupture or erosion causing thrombotic alterations that block blood flow to cardiac tissue [3]. The current gold standard treatment for AMI is percutaneous coronary intervention (PCI), a procedure whose objective is to restore the arrival of blood flow to the ischemic heart tissue [4]. Paradoxically, the restoration of blood flow to ischemic myocardial tissue induces additional damage. Studies in animal models of AMI have suggested that myocardial reperfusion is responsible for up to 50% of the final infarct size [3]. One of the key factors involved is oxidative stress, which influences myocardial reperfusion via multiple pathological mechanisms. Consequently, the antioxidant defense system could become overwhelmed, thus resulting in oxidative damage and, ultimately, cell death [5]. Numerous attempts have been performed to reduce myocardial tissue damage after ischemia-reperfusion (I/R) by enhancing the antioxidant defense system with antioxidant treatments. Accordingly, the administration of molecules such as vitamin C, vitamin E, N-acetylcysteine, deferoxamine and polyphenols, among others, has been studied [6]. However, these therapies have led to suboptimal and inconsistent results. As a consequence, until today, there is no reliable therapy against I/R damage for these patients. It still remains a challenge to find an effective therapeutic strategy that can prevent such damage and decrease infarct size after reperfusion by PCI.

Here, it is noteworthy that although antioxidant therapy has not been successful enough to be implemented, most of the studies have been mainly based on the administration of the antioxidants as monotherapies. Nevertheless, it is well known that the pathophysiology of cardiac tissue damage caused by I/R is multifactorial, and multiple forms of ROS generation might contribute to this damage. Therefore, as an alternative strategy, we should consider synergistic antioxidant combination therapies for multitarget effects with minimized toxicity and high selectivity [7]. Therefore, it would be expected that the search for new associations of antioxidants would lead us to achieve an improvement in the pharmacological response. 

To elucidate the translational potential of antioxidant therapies in the treatment of I/R injury after PCI, a detailed analysis of the available evidence with a focus on possible synergies between the different compounds is needed. Among the multiple natural available antioxidants, it has been evaluated the use of resveratrol, which is safe to use and effective on low doses, whose cardioprotective effect has been demonstrated in various studies performed in animal models, such as an I/R rat model, where a 21-day oral treatment with resveratrol decreased the incidence of atrioventricular block and lethality [8], and quercetin, which has been shown in various animal models to exhibit a variety of active biological functions, including anti-inflammatory, anticoagulation, and oxygen radical-scavenging activities as shown by the recent experiment in an animal model illustrating a reduction in the expression of various oxidant enzymes, thus decreasing oxidative stress [9].

### 1.1. Oxidative Stress 

#### 1.1.1. Oxidative Stress and ROS

Oxidative stress results from the imbalance between free radicals production and the antioxidant defense system [10]. Under oxidative stress conditions, two kinds of reactive species are produced, reactive oxygen species (ROS), such as radical anion superoxide, hydrogen peroxide, hydroxyl radical and reactive nitrogen species (RNS), which include nitric oxide radical, nitrogen dioxide radical, and peroxynitrite [11].

ROS can be formed by enzymatic or non-enzymatic mediated processes. Enzymatic ROS sources include reduced nicotinamide adenine dinucleotide phosphate oxidase (NADPH oxidase or NOX), xanthine oxidase (XO), uncoupled endothelial nitric oxide synthase (eNOS), among others [12]. In addition, an example of the non-enzymatic mechanisms of ROS production is the direct generation of hydroxyl radicals via Fenton and Haber–Weiss reactions, both of which depend on the availability of transition metal ions such as free iron in the form of labile iron pool (LIP). The hydroxyl radical is highly reactive and of low specificity, making it capable of damaging more biomolecules than any other ROS [13].

#### 1.1.2. Mechanisms of Ischemia/Reperfusion Cellular Damage 

**Figure 1:**
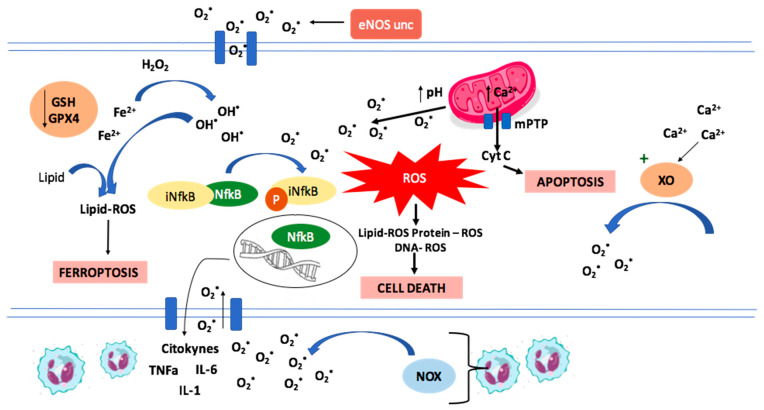
Representation of mechanisms involved in Ischemia/Reperfusion damage. The increase in intracellular calcium concentration is related to the activation of xanthine oxidase, a pro-oxidant enzyme. The increase in intramitochondrial calcium and the pH increase during reperfusion lead to the opening of mPTP and the loss of mitochondrial transmembrane potential, associated with ROS production. Reactive oxygen species activate the NFκB transcription factor, promoting inflammation and neutrophil migration to the injury zone and increasing ROS production by NADPH oxidase. Another ROS source is the uncoupled eNOS. Reactive oxygen species peroxidize lipids, proteins, and DNA, triggering cell death, together with apoptosis that happens from the release of cytochrome c through mPTP and ferroptosis that occurs in the context of a decrease in GSH and GPX4 activity, with accumulation of lipid peroxidation products.

The mechanisms accounting for I/R damage are depicted in Figure 1.

#### 1.1.3. ROS Production in Ischemia/Reperfusion

The occlusion of a coronary artery branch inhibits blood flow to myocardial tissue causing regional ischemia [14]. During myocardial ischemia, the decrease in the oxygen supply in cardiac tissue causes a marked reduction of oxygen supply to the mitochondrial electron transport chain, leading to a shift from aerobic to anaerobic cellular respiration. Consequently, a decrease in ATP production ensues, accompanied by lactic acid accumulation and a decrease in cellular pH [15]. The pH acidification increases the Na^+^ influx through the Na^+^/H^+^ exchanger, and the lack of ATP decreases the activity of the Na^+^/K^+^ pump, leading to an increase in intracellular Na^+^ that activates the Ca^2+^/Na^+^ exchanger to function in a reverse direction, thus exchanging intracellular Na^+^ with extracellular Ca^2+^ [16]. As a result, there is an overload in intracellular Ca^2+^ that cannot be captured by the Ca^2+^ ATPase (SERCA) in the sarcoplasmic reticulum due to the lack of ATP [17]. The overload of intracellular Ca^2+^ induces enzymes changes. One of them is the conversion of xanthine dehydrogenase in endothelial cells to xanthine oxidase (XO), an enzyme that generates superoxide anion radicals, contributing to the induction of oxidative stress [18]. Furthermore, during cardiac ischemia, tetrahydrobiopterin, a cofactor of endothelial eNOS, is oxidized, causing eNOS to get uncoupled and produce superoxide instead of nitric oxide. These changes are related to a burst in ROS production that has been found once reperfusion has restored oxygen supply to the myocardial tissue [19]. Exacerbation of oxidative stress during reperfusion overwhelms the endogenous antioxidant defenses, causing direct damage by lipid peroxidation, DNA oxidation and protein carbonylation, effects that can progress until cell death [19,20]. The main effectors of this damage are peroxynitrite and hydroxyl radicals generated from Fenton/Haber–Weiss reactions and [19].

Other ROS sources in cardiac tissue exposed to ischemia-reperfusion are NADPH oxidase in neutrophils, mitochondrial electron transport chain (mETC), cytochrome P450, lipooxygenase (LOX), cyclooxygenase, and monoamine oxidase [21].

### 1.2. Intracellular Organelle Dysfunctions Associated with Ischemia/Reperfusion Injury

#### 1.2.1. Endoplasmic Reticulum Stress (ERS) 

Endoplasmic reticulum stress has been described as another potential mechanism of I/R injury. It results from the accumulation of unfolded proteins in the endoplasmic reticulum, leading to an imbalance in calcium ion homeostasis and the activation of the unfolded protein response (UPR), a signaling pathway aimed to counteract the stress conditions effects otherwise leading to cell apoptosis [22]. Protein kinase-like ER kinase (PERK) is one of the sensors of stress that activate and coordinate the cell response following UPR activation. In particular, PERK may participate via the ERS-PERK signaling pathway in mediating the translocation and activation of the pro-survival nuclear factor erythroid 2-related factor 2 (Nrf2), suggesting that its influence on cell survival may be closely associated with regulating Nrf2/ARE signaling pathway [23].

#### 1.2.2. Mitochondrial Dysfunction

Mitochondrial permeability transition pore (mPTP) is a protein channel located in the inner mitochondrial membrane involved in necrotic and apoptotic cell death. When mPTP opens, it can induce the uncoupling of oxidative phosphorylation. Under ischemic conditions, the acid pH induced by lactate accumulation has an inhibitory effect on the mPTP, causing it to close [24]. Later, within the first few minutes of reperfusion [25], the intracellular acidic pH is restored to physiological values due to the reabsorption of lactate, and a mitochondrial Ca^2+^ overload ensues [26]. Furthermore, mPTP opens due to mitochondrial oxidative stress [27], uncoupling the oxidative phosphorylation and decreasing intracellular ATP availability, which can induce cell death through the mitochondrial pathway [26]. In addition, mPTP-induced cell death could be caused by the transport of molecules less than 1.5 kDa by mPTP, which collapses the mitochondrial membrane potential. Other consequences of mPTP opening are mitochondrial matrix swelling and the release of mitochondrial proteins, activating cell death mechanisms, such as apoptosis, necrosis, and necroptosis [28]. Moreover, the loss of mitochondrial potential generates a rise in ROS production [29].

#### 1.2.3. Inflammation Mediated Damage

The mechanism of I/R injury is usually considered sterile. However, it displays a series of inflammatory reactions resembling infectious processes such as inducing cytokines and chemokines production with infiltration of immune system cells [30,31]. This inflammatory response is thought to function as a double-edged sword: on one side, it enables the removal of cell debris and promotes wound healing. Damage-associated molecular pattern (DAMP) mediated signaling can also exacerbate the inflammatory state in a disproportional matter, thereby leading to additional tissue damage. After reperfusion, neutrophil and monocyte become activated by many damage signals released by necrotic cells. The primary immune response expands tissue damage by inducing neutrophil, NLRP3-inflammasome [32] and Toll-like receptor 9-pathogen recognition receptor activation [33], which converge on the activation of the myeloid differentiation primary response gene 88 (MyD88) and nuclear factor-κB (NF-κB) pathways [3] which in turn polarize T lymphocyte towards a pro-inflammatory phenotype. 

NF-κB proteins are a family of transcription factors that regulate the expression of genes involved in inflammation, immune response, cell proliferation, apoptosis [34] and activation of the mTOR pathway, leading to autophagy [35]. Reactive oxygen species can induce NF-κB activation through phosphorylation and degradation of NF-κB inhibitory cofactor IkBα, which allows NF-κB to translocate into the nucleus and modify cardiac tissue gene expression by up-regulation of genes involved in inflammatory and pro-fibrotic responses [36]. Neutrophils become important contributors to myocardial damage since they not only release proteolytic enzymes (hydrolases, metalloproteinases, and proteases) but also constitute a major ROS source to generate superoxide anions through NADPH oxidase (NOX) [37], causing damage to cardiomyocytes and endothelial cells. From a wound-healing perspective, after the inflammatory phase is dominated by pro-inflammatory T helper 1 lymphocytes, a second, anti-inflammatory reparative phase leading to wound healing and scar formation comes later. Cytokines have a key role in tissue repair. IL-6, IL-10, transforming growth factor-beta (TGF-β), and a subpopulation of T-lymphocytes known as ‘T Regulatory cells’ have all been associated with suppressing the pro-inflammatory response and steering the immune system towards repair and resolution following I/R [38,39]. Apoptotic neutrophils induce an anti-inflammatory phenotype in infiltrated macrophages upon their phagocytosis, which inhibits the macrophage proinflammatory tissue-damaging response and leads them to produce IL-10 and TGF-β [40,41], which dampening inflammation, as well as excessive tissue scarring during the reparative phase, thus inhibiting I/R-derived damage. 

### 1.3. Mechanisms of Cell Death in Ischemia-Reperfusion

#### Ferroptosis 

Ferroptosis is a significant mechanism of injury in myocardial infarction and can be a promising target to reduce infarct size [42]. Ferroptosis consists of an iron-dependent, non-apoptotic form of cell death [43]. It is a regulated form of necrosis that is driven by oxidative stress and is characterized by the accumulation of lipid peroxidation products and ROS derived from iron metabolism. 

Glutathione peroxidase 4 (GPX4) is the key regulator of ferroptosis [43]. This enzyme transforms phospholipid hydroperoxides to lipid alcohols using reduced GSH, which inhibits ferroptosis and explains why it mostly happens when cellular GSH levels are low, or GPX4 is inhibited [44]. Inhibition of GPX4 activity can lead to the accumulation of lipid peroxides, which is a marker of ferroptosis [43]. During I/R, intracellular reduced glutathione (GSH) decreases, as well as the activity of GPX4. Thus, lipid peroxides cannot be reduced by GPX4, and Fe^2+^ oxidizes lipids in a Fenton-like manner, resulting in a large amount of ROS concentration, which promotes ferroptosis.

Thus, iron metabolism is one of the main pathways of ischemia-reperfusion injury involved in cell death [42]. In animal models of coronary artery ligation-induced myocardial I/R injury, it has been observed that ferroptosis inhibition may repair cell damage and infarct size [43]. Iron is transported inside cardiomyocytes after recognition by the transferrin receptor 1 (TfR1) [45], and Ferritin (FT) is the main iron storage protein [13]. It is of interest to remark that ferrous ionic free iron is a key ferroptosis inducer; therefore, this deleterious process could be inhibited by iron chelators [46].

### 1.4. Apoptosis

Studies performed in in vivo animal models have demonstrated the important role of apoptosis in cell death following I/R, particularly during the phase of reperfusion [47]. Energy restoration characterizing reperfusion promotes the apoptotic process. Experiments in isolated rat hearts demonstrated that mitochondrial dysfunction and caspase activation occur after global ischemia and that caspase activation is dependent on the time of ischemia [48]. The opening of the mPTP and the changes in the mitochondrial transmembrane potential promote the release of pro-apoptotic proteins such as cytochrome c to the cytosol (23), leading to apoptotic cell death [49]. 

### 1.5. Defense Mechanisms against Oxidative Stress

Antioxidant systems regulate redox homeostasis by controlling the intracellular ROS levels and their interaction with biological molecules. This system has enzymatic and non-enzymatic mechanisms involved. Enzymes constitute the first line of antioxidative cellular defense; they are superoxide dismutase (SOD), glutathione peroxidase (GPX) and catalase (CAT). Non-enzymatic antioxidants can be exogenous or endogenous and include molecules such as vitamin C, vitamin E, reduced glutathione (GSH), carotenoids, flavonoids and polyphenols, among many others [50,51]. 

Antioxidants act by many mechanisms. Some of them are ROS scavenging or of their precursors, inhibiting ROS production, attenuating the catalysis of ROS generation via chelating metal ions, enhancing endogenous antioxidant generation, repairing the oxidative damage inflicted on the macromolecules and reducing apoptotic cell death by up-regulating the anti-apoptotic gene Bcl-2 [50]. Antioxidant enzymes are encoded by several housekeeping genes largely controlled by Nrf2 [52].

### 1.6. Nrf2 Signalling Pathway

Nrf2 is a transcription factor considered an essential regulator for maintaining the redox balance [53] via inducing endogenous antioxidant enzymes in response to oxidative stress [54]. Kelch-like ECH-associated protein 1 (Keap1) is a cullin3-dependent E3 ubiquitin ligation substrate [55] and a suppressor protein that physically binds Nrf2 and retains it in the cytoplasm so that it can be rapidly ubiquitinated and go to proteasomal degradation by the Cu3-Rbk1 complex [56]. Under oxidative stress conditions, intracellular ROS levels increase, and Keap1 undergoes conformational changes [57] that impair its ability to interact with Nrf2, thus facilitating the complex dissociation and preventing Nrf2 proteasomal degradation. Consequently, Nrf2 translocates into the nucleus and binds to the antioxidant response elements (ARE), located in the promoter region of target genes encoding antioxidant proteins, thereby enhancing their expression [36,58]. 

It is important to address that Nrf2 is activated by phosphorylation and that different molecules can do this from different pathways, such as phosphatidylinositol 3- kinase (PI3K) [59], protein kinase C (PKC) [60] and c-Jun NH2 terminal protein kinase (JNK) [61] pathways. Meanwhile, glycogen synthase kinase 3β (GSK3β) promotes Nrf2 degradation independently from keap1 and Nrf2 translocation and inactivation in the nucleus [62]. Nrf2 participates in the regulation of cellular defense and mediates cellular repair, proliferation, and/or regeneration. In addition, Nrf2 protects the mitochondria against oxidative damage [63] and tissues from harm, exerting anti-inflammatory and anti-apoptotic effects [53]. As for the anti-apoptotic effect, Nrf2 is involved in the up-regulation of uncoupling protein (UCP)3 expression by binding to its promoter, and this signaling mechanism promotes cell survival under oxidative stress [54]. UCPs are inner membrane proteins of mitochondria that regulate the uncoupling of oxidative phosphorylation and protect against pro-oxidant mediators [64].

Nrf2 signaling may also be involved in autophagy regulation. Recent research shows an increased Nrf2 nuclear expression and a decreased cytosolic concentration in mice treated with rapamycin, a known autophagy inducer. Moreover, Nrf2-KO mice treated with rapamycin after AMI had increased levels of apoptosis, oxidative stress, and a bigger infarct size when compared to regular mice treated with rapamycin [65]. 

In summary, Nrf2/Keap1/ARE corresponds to a relevant signaling pathway that can attenuate myocardial infarct size after myocardial ischemia and reperfusion by up-regulation of antioxidant, anti-inflammatory, and autophagy mechanisms, which leads to a cardioprotective effect.

### 1.7. Exogenous Antioxidants with Reported Cardioprotective Effects

**Figure 2:**
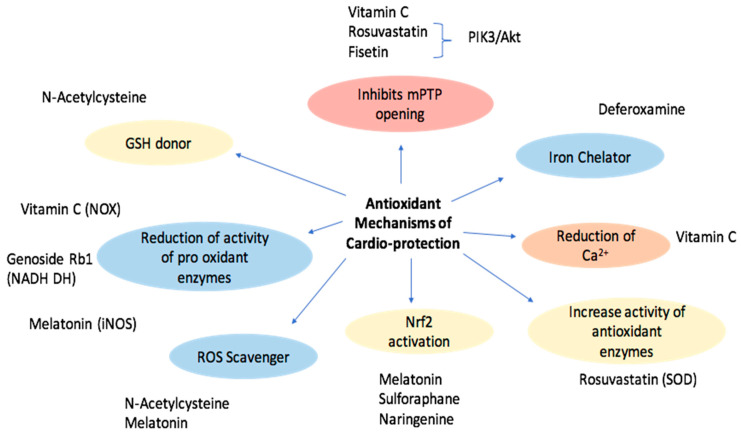
Schematic representation of the different cardioprotective effects of antioxidants and related molecules.

The potential usefulness of antioxidants as protective tools against I/R damage has attracted enormous interest. Clinical and experimental studies have been performed to determine the ability of natural or synthetic compounds with antioxidant and anti-inflammatory properties to counteract the mechanisms responsible for tissue alteration during I/R and to identify the involved molecular pathways [66]. Table 1 and Table 2 and Figure 2 summarize the molecules evaluated with respect to their cellular protective mechanisms.

### 1.8. Resveratrol

**Figure 3:**
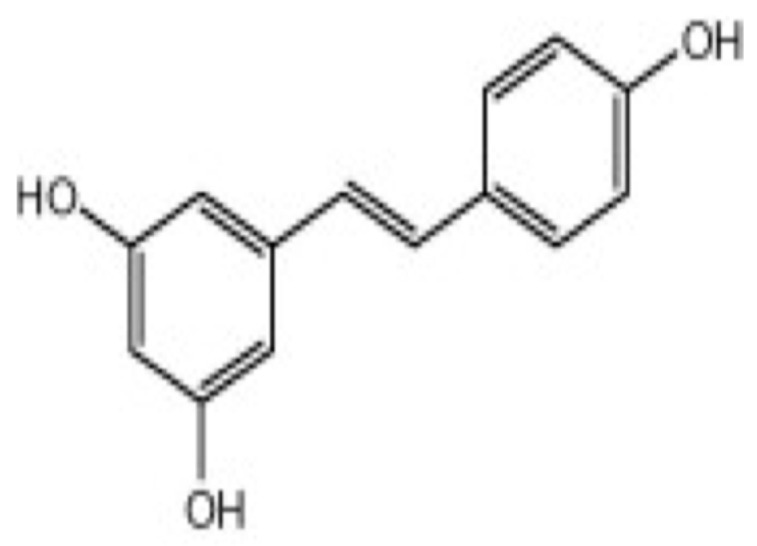
Trans resveratrol Structure (3,5,4′-trihydroxy-trans-stilbene).

Resveratrol is a natural polyphenol with multiple biological activities [90]. It has a molecular weight of 228.25 g/mol [8] and exists in two isomeric forms (cis and trans), with the trans form (Figure 3) being the most common with more therapeutic benefits [91]. This antioxidant is found in many fruits and vegetables, such as grapes, *Polygonum cuspidatum* and mulberry [92]. 

Resveratrol became of interest during the early 1990s when a French paradox study mentioned that it was beneficial for health [91]. Since then, this molecule has been the subject of numerous studies, and many physiological effects have been assigned to it. Different studies have evidenced that it has various properties, including anti-inflammatory, antioxidant, anti-diabetic, antihypertensive, anti-cancerous and cardioprotective effects [90,92]. 

### 1.9. Resveratrol Cardioprotective Mechanisms 

Regarding its cardioprotective action, several mechanisms have been designed attempting to understand which molecular pathways allow resveratrol to have an impact on cellular conditions related to myocardial damage due to I/R, such as oxidative stress, apoptosis, autophagy, hypoxia-induced endoplasmic reticulum stress, nitric oxide synthesis and others [93,94]. 

**Figure 4:**
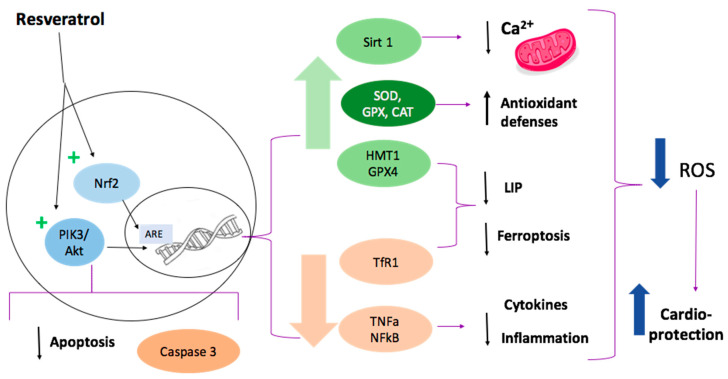
Representation of resveratrol cardioprotective effect. Resveratrol activates the Nrf2 signaling pathway, increasing the expression of antioxidant enzymes. It also reduces the expression of pro-apoptotic proteins, such as caspase 3, via the PI3K/Akt pathway. Other effects are the reduction of inflammation and ferroptosis, which has been associated with reduced expression of TNFα, Nf*κ*B, TfR1, and increased activity of GPX4 after administration of resveratrol. The activation of SIRT1 is related to a decrease in intramitochondrial calcium concentration, which diminishes mitochondrial ROS production.

We will discuss some of the molecular pathways that have been described as the potential for resveratrol effect on cardiomyocytes. It is relevant that most studies have been performed in rat myocardial in vivo I/R models (Figure 4). 

#### 1.9.1. SIRT1 Activation

Silent information regulator factor 2-related enzyme 1 (SIRT1) consists of a nicotinamide adenine dinucleotide-dependent deacetylase that regulates oxidative stress-related proteins and other proteins that affect the function of cardiomyocytes. It was proposed that resveratrol activates SIRT1 leading to the activation of a cardioprotective response, reducing the Ca^2+^ overload in the cytosol and mitochondria [95]. According to this theory, a recent study showed that in a rat model of cardiac I/R, 21-day oral treatment with resveratrol decreased the incidence of atrioventricular block and lethality, thus suggesting the usefulness of this natural compound in the prevention of lethal cardiac arrhythmias after reperfusion [90]. Furthermore, it has been demonstrated that intraperitoneal injection of resveratrol in mice determines the increase of SIRT1 levels and a concomitant decrease of UCP-2 expression, thus protecting myocardial tissue against I/R injury in vivo [96].

#### 1.9.2. Ferroptosis Inhibition

In a recent study carried out by Li et al. [89], in vitro experiments with H9c2 cells and a model of oxygen-glucose deprivation/reoxygenation (OGD/R) demonstrated that OGD/R-induced H9c2 cells showed increased cell death by ferroptosis. The addition of resveratrol in cell cultures reduced oxidative stress and the content of Fe^2+^. In vivo experiments performed by the same authors in rats ligated and perfused by the left anterior descending branch showed that when resveratrol was administered at the concentration of 50 mg/kg for 14 days, oxidative stress and the content of Fe^2+^ decreased again. The effect was explained by changes in the regulation of gene expression, in which resveratrol down-regulated transferrin receptor 1 expression and up-regulated the expression of ferritin heavy chain 1 and GPX4, leading to inhibition of ferroptosis and potentiation of the antioxidant defenses, respectively. The consequent reduction in oxidative stress is evidenced by a reduction in malondialdehyde (MDA) production [89].

#### 1.9.3. Attenuation of Inflammation and Apoptosis

There are multiple ways in which resveratrol might inhibit apoptosis. Yu et al. [97] studied rats the antioxidant and anti-apoptotic effects of resveratrol in myocardial I/R injury. Compared to the control, the resveratrol group showed decreased ROS levels, increased catalase and glutathione peroxidase activities, and increased cell viability by apoptosis inhibition. Further, a decrease in lactate dehydrogenase (LDH) and plasma creatine kinase MB (CK-MB) levels were found. They suggested that these effects were mediated by the activation of the PI3K/AKT signaling pathway, which down-regulates the expression of apoptotic genes and regulates cell proliferation [98,99]. Apparently, PI3K/AKT signaling pathway is inhibited during the I/R process, resulting in potent activation of apoptosis and less viable tissue [97,100]. Moreover, it has been suggested that resveratrol attenuates apoptosis by reducing caspase 3 expression [101]. In another study, Hu et al. [102] indicated that resveratrol attenuates necroptosis after ischemia-reperfusion by inhibition of the tumor necrosis factor-alpha (TNF-α)/receptor-interacting protein kinase 1 (RIP1)/RIP3/mixed-lineage kinase domain-like (MLKL) signaling pathway. The results were that after treatment with different resveratrol concentrations, the expressions of TNF-α, RIP1, RIP3, and p-MLKL/MLKL decreased, as well as necroptosis. Under those circumstances, cell viability increased. All these effects were seen in dose-dependent manners [102]. Xing et al. [103] measured the levels of serum interleukin-1*β* (IL-1*β*), IL-6, and TNF-*a* in rat myocardial tissue during I/R injury. They also measured the expressions of mRNA and proteins of Toll-like receptor 4 (TLR4), NF-*κ*B, p65, IL-1*β*, IL-6, and TNF-*α*. Resveratrol treatment resulted in a significant reduction of serum IL-1*β*, TNF-*α*, and IL-6, TLR4, NF-κB, p65, IL-1*β*, IL-6, and TNF-*α* mRNA and protein expressions in cardiac tissue cells [103].

#### 1.9.4. Nrf2 Activation

As indicated before, Nrf2 up-regulates the expression of proteins involved in the antioxidant response. Using an in vitro hypoxia–reoxygenation (HR) model on HUVEC, it has been demonstrated that the increased cell viability and the reduced apoptotic rate and oxidative stress observed in cells treated with resveratrol were significantly reversed by knocking down Nrf2 [104]. Furthermore, in a rat model of lower-extremity I/R, treatment with resveratrol improved the apoptotic state of the femoral artery and reduced oxidative stress; this effect was abolished by the co-administration of an inhibitor of Nrf2 [104]. These results indicate that resveratrol might attenuate the I/R injury damage through up-regulating Keap1/Nrf2 signaling, mainly for its effects on oxidative stress. This could increase cell viability and decrease the apoptotic rate [104]. 

#### 1.9.5. Reduced Mitochondrial Dysfunction 

A recent study demonstrated that the addition of resveratrol in an in vitro I/R model obtained with primary rat cardiomyocytes decreased lactate dehydrogenase and creatine kinase MB release and ROS production and increased cell viability and catalase and glutathione peroxidase activities [97]. Moreover, resveratrol significantly increases the activity of mitochondrial superoxide dismutase and reduces the levels of malondialdehyde, indicating reduced oxidative damage to the mitochondria. The effects observed after resveratrol addition were reversed using PI3K siRNA, thus suggesting that the protective effect of resveratrol on cardiomyocytes under I/R conditions can be due to PI3K/AKT signaling pathway activation [97].

#### 1.9.6. Other Pathways

It has also been proposed that resveratrol could reduce oxidative stress and protect myocardial cells via activating the VEGF-β/antioxidant signaling pathway [105]. Particularly, experiments conducted on rat hearts and on H9c2 cells showed that after treatment with resveratrol, a marked improvement of left ventricular function and a reduction of infarct size *ex vivo*, and decreased cell death and apoptosis of H9c2 cells during I/R occurred. The treatment with resveratrol was associated with the up-regulation of VEGF-*β* mRNA and protein levels, which caused the activation of Akt and the inhibition of GSK3β. The inhibition of VEGF-*β* inhibited the cardioprotective effects of resveratrol [105]. Of interest, the treatment of cardiomyocytes with isorhapontigenin (ISO), a resveratrol analog, was able to counteract angiotensin (Ang) II-induced cardiac hypertrophy by inhibiting Ang-II-dependent phosphorylation of PKC, Erk1/2, JNK, and p38. The same authors also observed that pretreatment with ISO down-modulated Ang II-mediated NF-κB activation by affecting the degradation and phosphorylation of IκBα [106]. 

#### 1.9.7. Considerations of Safety in the Use of Resveratrol

Resveratrol is one of the potential therapeutic agents for the prevention of I/R heart tissue damage, but there are some issues that must be addressed. Shaito et al. [91] reviewed the potential adverse effects of the use of resveratrol. Resveratrol showed a beneficial effect at low doses, and the optimal dosage with safer and higher potency is still under research. Resveratrol presents a hormetic effect, and high doses have toxic effects, including inhibition of p450 cytochromes, which leads to interaction with several drugs and pro-oxidant effects [107]. We should pay attention to this dose-dependent effect on the redox state (antioxidant at low doses and pro-oxidant at high ones), given the fact that we are focused on an antioxidant activity [91].

Furthermore, studies usually focus on the short-term outcomes of resveratrol administration. Therefore, long-term effects are widely unknown. Additionally, there is not enough information about interactions between resveratrol and other therapies and little research about its pharmacokinetics, especially regarding absorption and bioavailability [91]. Referring to resveratrol water solubility, it is 0.03 mg/mL [108], which leads to low bioavailability. Nevertheless, different formulations have been designed to overcome this limitation, which will be discussed below. 

#### 1.9.8. Associations between Resveratrol and Other Antioxidants

Different pathways and mechanisms participate in the generation of I/R injury. Even though resveratrol interferes with many of those mechanisms to reduce and/or prevent I/R damage, others are not covered by it. Therefore, it is reasonable to think that it would be beneficial to propose an association of resveratrol with other natural antioxidants (Table 1), looking for a synergic effect able to reduce the damage as much as possible. Until now, associations of resveratrol with other antioxidants or clinical drugs have not been studied. As a result, in the search for the most beneficial multitarget therapeutic scheme, we propose that there could be a synergistic effect between vitamin C and deferoxamine. 

Resveratrol is associated with activating the Nrf2 signaling pathway, which leads to an increased expression of antioxidant enzymes, potentiating the antioxidant response. Theoretically, the balance would be even more inclined to favor this response if we decrease the function of pro-oxidant enzymes. In this context, adding vitamin C would be beneficial as it down-regulates NADPH oxidase activity and prevents eNOS uncoupling [50]. Further, it reduces intracellular Ca^2+^ overload and prevents the depolarization of the mitochondrial membrane [67]. These two last effects are related to SIRT1 activation by resveratrol, while it has been seen in studies showing that vitamin C also increases the expression of SIRT1 [109]. Even if this could constitute a potential interference between both antioxidants, it is necessary to look further into the molecular mechanisms involved to determine whether it would potentiate or antagonize the effect of resveratrol. It is worth mentioning that vitamin C is a water-soluble vitamin and can be administered intravenously [110]. 

### 1.10. Quercetin 

**Figure 5:**
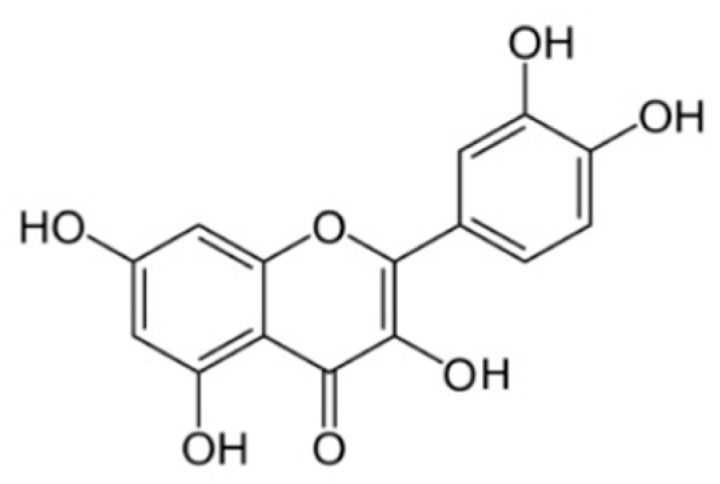
Quercetin Structure (3,30,40,5,7-pentahydroxyflavone).

Quercetin (Que) is a flavonoid with the following chemical formula C_15_H_10_O_7_ (Figure 5) [111].

**Figure 6:**
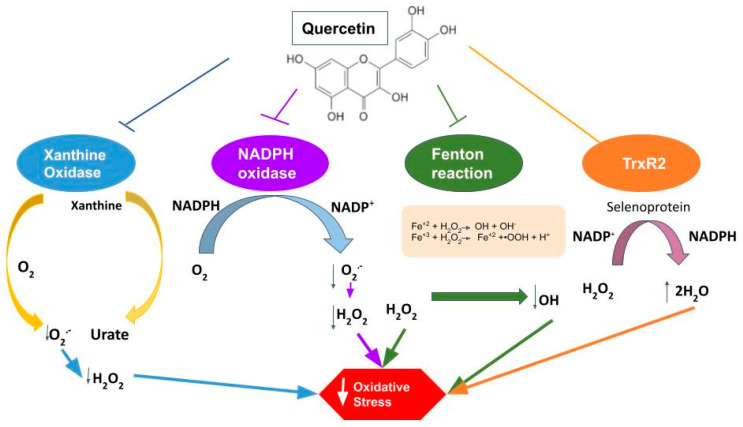
Representation of quercetin cardioprotective effect. Quercetin acts through the inhibition of the enzymes xanthine oxidase, NADPH oxidase and the Fenton reaction, mechanisms leading to a decrease in ROS production. In addition, it acts by activating the selenoprotein TrxR2, which has a powerful antioxidant effect by inactivating ROS. All these mechanisms lead to a decrease in oxidative stress.

Flavonoids are compounds with very different conformations. They are hydrophobic, as they have two benzene rings and a pyran ring between them [112]. Flavonoids exist in most plants and account for 65–75% of our daily flavonoid intake [113], mainly in vegetables and fruits, and also have a strong antioxidant property attributed to the presence of five hydroxyl groups, together with the pyrocatechol, which makes them good scavengers of free radicals [114]. Unfortunately, despite being very promising, Que is a molecule with low bioavailability due to its low aqueous solubility. However, many efforts have been devoted to increasing its solubility to obtain analogs with potentially improved properties. Quercetin can be rapidly hydrolyzed in the digestive tract by the enzyme β-glucosidase, which will facilitate its absorption through the intestinal mucosa, to be finally transferred to all the rest of the organism through portal circulation [115]. It also has several other biological effects in addition to its antioxidant properties. It has anti-aggregating, anti-inflammatory, anti-cancer, and anti-aging effects. Furthermore, it is not toxic even in high doses (4000 mg/day) [116]. Additionally, recent studies have shown it to have tremendous potential to reduce myocardial damage that occurs after I/R through various mechanisms (Figure 6) [117].

#### 1.10.1. Properties of Quercetin against Oxidative Stress 

Recent experiments with an animal model of hyperuricemia have demonstrated a reduction in xanthine oxidase expression and enzyme activity by Que treatment (100, 200, 400 mg/Kg) [9]. Xanthine oxidase is a molybdo-flavoenzyme found in various species. It is a homodimer with two symmetrical monomers. It has a great affinity for producing ROS because, in each of the monomers, there is a C-terminal molybdo protein that contains four redox centers. In turn, it has an N-terminal domain with two iron–sulfur centers and a central flavin adenine dinucleotide cofactor [118]. Thus, xanthine oxidase stimulates ROS production, which can end up causing oxidative stress injuries [119]. 

Similarly, Que can attenuate the expression of NOX2 [120]. The NADPH oxidase system is a multiprotein complex that produces ROS in different cells and tissues, being of great importance in phagocytic cells [121]. The NADPH oxidase enzyme is composed of several subunits, including the gp91-phox subunit and its counterparts which are commonly known as the NOX family (NOX1, NOX2, NOX3, NOX4, NOX5, DUOX1, and DUOX2) [122] that are found in almost all cells of the organism [123]. In the cardiovascular system, this complex is the main ROS producer, which is achieved through the transfer of an electron from NADPH to O_2_, resulting in NADP and O_2_^●−^. The newly produced O_2_^●−^ will end up rapidly transforming into H_2_O_2_, characterized by being more stable and diffusible [124]. This leads us to think that if we initially inhibit the expression of the enzyme, we will reduce the production of H_2_O_2_ and, consequently, decrease the I/R damage produced by ROS in the myocardial tissue. In cardiomyocytes, NADPH oxidase can be activated by various mechanisms and stimulating factors [125]. Among them, one of the stimulants for the enzyme corresponds to I/R, with NOX2 and NOX4 being the ones more activated during myocardial I/R injury [82]. Therefore, the inhibitory effect of Que on NOX can potentially be a great target to ameliorate the injury induced by I/R. 

In addition, Que combined with Fe^2+^/Cu^+^ will significantly inhibit Fenton reaction, another ROS source producing ^●^OH [126]. Fenton reaction consists of the electron transfer from Fe^2+^/Cu^+^ metallic ion to H_2_O_2_, which produces Fe^3+^/Cu^2+^, OH− and ^●^OH. It has been shown that ^●^OH has a very strong oxidability in acid solutions [127]. Then also, H_2_O_2_ can restore Fe^2+^/Cu^+^ from Fe^3+^/Cu^2+^, which produces HOO^●^ and H^+^. Fe^2+^/Cu^+^ can be oxidized by O_2_ again, which forms O_2_^●−^. In I/R damage, an acidic environment is created due to anaerobic glycolysis, which then facilitates the production of the Fenton reaction. 

Selenium is an antioxidant and a component of selenoproteins, including glutathione peroxidases (GPX), thioredoxin reductases (TrxR) and methionine sulfoxide reductase 2, thereby modulating redox activity [128]. Accordingly, it has been observed that after Que administration at a dose of 50 mg/kg for eight weeks, the selenoprotein TrxR2 increased in the Que group compared to the control [82].

Quercetin is recognized as a significant antioxidant, preventing damage to cardiomyocytes as measured by oxidative stress, avoiding ROS increase and progression of damage after I/R. Moreover, ROS removal may increase NO bioavailability and restoration of endothelial function after I/R damage [129]. The mechanisms whereby Que exerts its antioxidant activity consist of eliminating ROS through its ability to react with the free radicals O_2_^●−^, HO^●^, NO, alkoxy and peroxyl. It also occurs when the hydroxyl attached to the benzene ring transfers a hydrogen atom or an electron to the free radical, resulting in the formation of more stable molecules [130]. Quercetin also acts through indirect mechanisms, restoring inherent antioxidant systems of the body, such as enzymatic systems dependent or not on antioxidants, such as vitamins C and E, and reduced glutathione (GSH) [59], the latter decreasing after I/R injury, which indicates the depletion of the antioxidant system. Thus, it can eliminate ROS and restore the function of the intrinsic antioxidant system [131].

#### 1.10.2. Anti-Inflammatory and Anti-Apoptotic Effects 

Acute inflammatory reactions are intensified in cardiomyocytes following I/R injury. This is due to the neutrophilic granulocytes, which are the ones that explain most of the reduced exudation and even the diffusion of these neutrophils through the blood vessels. Thus, it can be generated more damage to the injured tissue [132]. In the I/R period, leukocytes can alter the function of endotheliocytes and reduce the relaxation capacity of the coronary arteries [133]. They also promote coagulation and pro-inflammatory effects that further aggravate the mechanical obstruction of capillaries. This will cause a decrease in blood flow and incomplete perfusion of the coronary arteries, which are the main causes of neutrophil conglutination in the vascular walls [134]. The binding of leukocytes to endotheliocytes plays an important role in the inflammatory reaction, which is accompanied by cardiomyocyte apoptosis. Increased permeability of the plasmalemma leads to cytochrome C release that further aggravates myocardial apoptosis [135]. In this context, Que has been shown to improve inflammation and apoptosis in the myocardium. A recent I/R injury model was made through the ligation of the left coronary artery, after which Que was administered (2, 10 and 20 mg/kg orally) and diltiazem (15 mg/kg orally) for five days. This treatment caused the inhibition of the expression of TNF-α, IL-6 and IL-1β (important biomarkers of inflammatory reactions) in serum and cardiomyocytes. This indicates that Que treatment after I/R can alleviate inflammation and apoptosis. In agreement with these findings, Que diminishes the infarct size, as shown with 2,3,4-triphenyltetrazolium chloride (TTC) staining, as well as myocardial contractility, demonstrating that Que is capable of mitigating the inflammation and restoring myocardial function [131]. 

#### 1.10.3. Vasodilatory Effects 

It is known that during a period of ischemia, the coronary arteries are usually obstructed by emboli. Although blood flow can be quickly restored through interventional therapies, there are multiple findings that show that G-protein-coupled receptors are altered after I/R injury [136]. The administration of Que has been shown to improve vasoconstriction caused by ET-1, one of the types of endothelin, which is one of the most potent vasoconstrictors distributed in the endothelium of blood vessels (as well as in many other tissues and cells) [137].

#### 1.10.4. Associations between Quercetin and Other Antioxidants

Although Que acts by reducing oxidative stress, there are many mechanisms enhancing I/R damage that are still functioning. Therefore, it is proposed to associate Que with other antioxidants to produce synergy and further reduction of oxidative damage. There are multiple possible associations between Que and other antioxidants that could enhance the cardioprotective effect. One of the associations that have been studied is the combination between Que and α-tocopherol, which was studied in rats that were induced AMI by isoproterenol and were previously administered a combination of Que (10 mg/Kg) and α-tocopherol (10 mg/Kg) for 14 days. The combined pretreatment normalized all biochemical parameters and minimized ECG alterations. Therefore, Que and α-tocopherol exhibited enhanced cardioprotective effects against isoproterenol-induced cardiotoxicity because they eliminate free radicals, improve antioxidation, and maintain Ca^2+^ levels. Furthermore, the study showed that the combined pretreatment was more effective than the sole one [138]. There are other molecules with which the association with Que has been studied to demonstrate its cardio-supporting effect, such as lycopene, another natural phytocompound with antioxidant effects. In an animal model study in which cardiac toxicity was induced by isoproterenol (ISO), free radicals and oxidative stress increased in myocardial tissue. The combination of Que with lycopene prevented all the side effects of cardiotoxicity, significantly decreasing the myocardial damage, reducing oxidative stress, and slowing the levels of expression of antioxidant genes, a participant in pathways related to the Nrf2, HO-1, NQO1, GSTµ, SOD, SOD2, CAT and BCL-2 genes [139]. Evidence of the association of quercetin with clinical drugs was not found. 

## 2. Discussion

Oxidative stress constitutes an essential mechanism of damage involved in I/R injury in acute myocardial infarction. Even if multiple individual antioxidants have been tested to prevent or minimize this damage, an effective treatment is not available. This problem might be related to the fact that oxidative stress results from different pathways that lead to an imbalance between ROS generation and antioxidant activity. Some of these mechanisms are mitochondrial dysfunction, activation of the inflammatory response, decreased antioxidant activity and activation of pro-oxidant enzymes, such as xanthine oxidase, uncoupled eNOS, NOX, and others. The comprehension of oxidative stress mechanisms makes us realize that the treatment alternatives based on monotherapy are not the appropriate solution, a view that has not been changed for more than a decade. It is of interest that several isolated beneficial effects could be joined to improve the cardioprotective effects occurring in I/R settings, such as percutaneous coronary intervention following AMI. Thus, some antioxidants are iron chelators (deferoxamine), some are ROS scavengers (N-acetylcysteine), some activate the Nrf2 antioxidant response pathway (naringenin, melatonin, sulforaphane), and others exert influence on alternative edges of ROS production, resulting in effects that could be synergic and beneficial (Table 1). In the case of resveratrol and quercetin, these molecules have several cardioprotective effects making them suitable to be included as part of a multitarget therapy. 

It is important to note that resveratrol and quercetin molecular sites of action are still not fully elucidated, but there are many proven targets. In the case of resveratrol, activation of Nrf2 and SIRT1 occurs through increased antioxidant proteins expression and PGC-1α deacetylation [140]. In addition, resveratrol also activates the PI3K/AKT pathway during I/R injury, resulting in decreased mitochondrial pathway-mediated apoptosis [97]. As mentioned, resveratrol is also capable of reducing ferroptosis by decreasing Fe^2+^ concentration, which could be involved in inducing changes in the regulation of gene expression, but the exact target remains to be fully elucidated.

On the other hand, quercetin exerts its protective effects mainly through the inhibition of ROS-producing enzymes, such as NADPH oxidase and xanthine oxidase. The exact mechanism whereby this is achieved has been only partially described. A recent study shows that NOX may be inhibited for the enhanced expression of the antioxidant enzyme heme oxygenase-1 (HO-1) by quercetin [141]. In the case of xanthine oxidase, quercetin acts as a reversible inhibitor of the enzyme action [142].

Another relevant aspect is that there are some concerns about the water solubility of these compounds, which could make us question whether the suitability can be successfully administered intravenously. To solve this problem, different strategies have been designed to increase their water solubility. In the case of resveratrol, showing cardioprotective efficacy at low concentrations, it has been reported its administration with complexes with β-cyclodextrin and hydroxypropyl-β-cyclodextrin [143], complexes that could slow down resveratrol metabolism and increase bioavailability [144]. Furthermore, solubilization of resveratrol could be increased with micellar solutions of bile acids, an effect attributed to the smallest membranolytic potential achieved through the formation of bile acids derivatives with resveratrol [145]. On the other hand, multiple studies aim to increase Que solubility by various mechanisms and thus increase its application properties. One of them is the nanoformulation method, which improves solubility, bioavailability, and circulation time, among other effects [146]. In turn, Que solubility and its thermal degradation in ethanol expanded with CO_2_ and ethyl lactate were studied [147]. On the other hand, co-crystallization has recently gained attention as a means of improving the physicochemical characteristics of a compound, and each of these cocrystals exhibited pharmacokinetic properties that are far superior to those of Que alone [148]. Finally, all these solutions sustain the research that has been done about these two antioxidants and their potential cardioprotective effects. The improvement of solubility with different agents makes it possible to continue considering resveratrol as a suitable potential part of therapy against I/R injury. The evidence leads us to think that if the solubility of Que is increased, its therapeutic effects could be promisingly improved.

## 3. Concluding Remarks

Ischemia/reperfusion injury occurring in AMI patients undergoing PCI is a complex process involving pathophysiological cascades leading to cellular disturbances ranging from metabolic changes to cell death. Although oxidative stress is involved in several of these injury mechanisms, antioxidant therapeutic interventions so far have not been successful in protecting myocardial tissue against the harmful effects. Likely, the use of the most frequent monotherapies in a multifactorial process could explain the lack of consistency between the experimental studies and the clinical data. The paradigm of a multitarget therapy based on an association of different antioxidant molecules should be expected to improve the abrogation of oxidative damage and related effects derived from ROS burst, particularly occurring early at the onset of reperfusion. Thus, the administration of several antioxidant molecules could give rise to an additive or synergistic pharmacological effect. Some compounds, such as N-acetylcysteine, ascorbate and deferoxamine, have been tested as monotherapies in other clinical settings associated with increased ROS, showing lightly protective effects, as well as those caused by other naturally occurring antioxidant molecules. Among the latter, cardioprotective properties have been found in the case of resveratrol and Que, among others, having efficacy at low concentrations suitable to avoid adverse events. Therefore, these compounds could also be considered in this association, but following a better characterization of their pharmacokinetic properties, solubility at the required doses and studies about the appropriate design of formulations. Studies about associations of these compounds with others that have previously shown cardioprotective effects could be analyzed in isolated heart rat Langendorff model and, subsequently, in clinical trials aimed to reduce the infarct size due to the increased efficacy of this therapeutic association. This could lead to a better prognosis and quality of life after an acute myocardial infarction, decreasing complications, such as heart failure and arrhythmias, and also reducing the associated costs.

The evidence supports that resveratrol is safe to be used in low doses. It has strong antioxidant effects, in which Nrf2 and SIRT1 activation are involved, thereby increasing the antioxidant response and decreasing inflammation and ferroptosis. Taken together, these effects could be beneficial as part of a multitarget therapy. Nevertheless, further studies are still lacking to implement a therapeutic scheme that includes resveratrol involving association with other antioxidants, specifically with vitamin C, which inactivates the NOX, reducing ROS production, and the iron chelator deferoxamine, reducing ferroptosis and cell death. We propose resveratrol might show a synergic effect with these antioxidants due to a theoretical potentiation of oxidative stress reduction. Quercetin has multiple beneficial effects, particularly through its antioxidant activity could account for an enhanced cardioprotective effect. 

To date, the association of resveratrol and other antioxidants has not been studied, as well as there is no evidence of the combination of resveratrol or quercetin with clinical drugs. This is a topic that could be interesting to investigate, considering the beneficial and antioxidant properties of some drugs, such as melatonin, rosuvastatin and N-acetylcysteine, that are exposed in Table 2. In this case, being synthetic antioxidants, it would be important to consider what happens with the side effects of the drug in use.

Even if treatment for I/R injury has not been found yet, resveratrol and quercetin constitute attractive natural pharmacological agents to be used in a combined therapy aimed to reduce cardiac tissue damage and infarct size due to their cardioprotective effects. Randomized double-blind clinical trials should be made to test the therapeutic efficacy as well as safety of these associations. 

## Figures and Tables

**Table 1:** Natural Antioxidants.

Molecule	Cellular Mechanisms	Main Effects	References
Vitamin C	Reduction of ROS burst Down-regulation of NOX activity and prevention of eNOS uncoupling Reduction of intracellular Ca^2+^ overload Prevention of mitochondrial membrane potential depolarization	Antioxidant	[36,67]
Deferoxamine	Iron Chelation	Antioxidant	[68,69]
Curcumin	Activation of JAK/STAT3 signaling pathway	Anti-inflammatory Anti-apoptotic Antioxidant	[70,71]
Ginsenoside Rb1	Inhibition of ROS production in mitochondrial complex l (NADH dehydrogenase) Possible activation of Anti-apoptotic mTOR pathway Increasing GSH and NO expression	Antioxidant Anti-inflammatory Anti-apoptotic	[72,73,74]
Fisetin	Activation of PI3K/Akt/GSK-3β pathway Reversion of mitochondrial dysfunction	Antioxidant	[75]
Naringenin	Regulation of Nrf2 pathway and inhibition of ferroptosis	Ferroptosis Inhibitor	[76]
Sulforaphane	Regulation of Nrf2 pathway	Antioxidant	[77,78]

**Table 2:** Synthetic antioxidants.

Molecule	Cellular Mechanisms	Main Effects	References
N-Acetylcysteine	GSH donation Increase in microvascular blood flow ROS Scavenger	Antioxidant Anti-inflammatory	[79,80,81]
Metformin	AMPK-dependent NOX4 suppression and eNOS activation Suppression of NLRP3 inflammasome activation	Antioxidant Anti-inflammatory Anti-apoptotic	[82,83,84]
Rosuvastatin	Activation of Akt and GSK-3*β* Enhancement of superoxide dismutase activity Inhibition of mPTP opening Up-regulation of PPAR-γ and UCP2	Antioxidant Anti-inflammatory	[85]
Dexmedetomidine	Down-regulation of ERS signaling pathways (PERK, CHOP, IREI) Increase of NO production via PI3K signaling	Anti-inflammatory Anti-apoptotic	[86,87,88]
Melatonin	Free radical scavenging Activation of Nrf2 pathway Reduction of NFκ*B* binding to DNA Inhibition of iNOS and cyclooxygenase expressions	Antioxidant Anti-inflammatory	[89]

## Data Availability

Not applicable.
